# Ionic Strength‐Mediated “DNA Corona Defects” for Efficient Arrangement of Single‐Walled Carbon Nanotubes

**DOI:** 10.1002/advs.202308532

**Published:** 2024-01-17

**Authors:** Yuanyuan Luo, Na Wu, Liqiong Niu, Pengyan Hao, Xiaoya Sun, Feng Chen, Yongxi Zhao

**Affiliations:** ^1^ Institute of Analytical Chemistry and Instrument for Life Science The Key Laboratory of Biomedical Information Engineering of Ministry of Education School of Life Science and Technology Xi'an Jiaotong University Xianning West Road Xi'an Shaanxi 710049 China; ^2^ Frontier Institute of Science and Technology Xi'an Jiaotong University Xi'an Shaanxi 710049 China

**Keywords:** carbon nanotubes, DNA corona phase, DNA nanotechnology, DNA origami, self‐assembly

## Abstract

Single‐stranded DNA oligonucleotides wrapping on the surface of single‐walled carbon nanotubes (SWCNTs), described as DNA corona, are often used as a dispersing agent for SWCNTs. The uneven distribution of DNA corona along SWCNTs is related to the photoelectric properties and the surface activity of SWCNTs. An ionic strength‐mediated “DNA corona defects” (DCDs) strategy is proposed to acquire an exposed surface of SWCNTs (accessible surface) as large as possible while maintaining good dispersibility via modulating the conformation of DNA corona. By adjusting the solution ionic strength, the DNA corona phase transitioned from an even‐distributed and loose conformation to a locally compact conformation. The resulting enlarged exposed surface of SWCNTs is called DCDs, which provide active sites for molecular adsorption. This strategy is applied for the arrangement of SWCNTs on DNA origami. SWCNTs with ≈11 nm DCD, providing enough space for the adsorption of “capture ssDNA” (≈7 nm width required for 24‐nt) extended from DNA origami structures are fabricated. The DCD strategy has potential applications in SWCNT‐based optoelectronic devices.

## Graphical abstract

Ionic strength‐mediated phase transition of wrapping DNA on the surface of single‐walled carbon nanotubes (SWCNTs) is applied to obtain enlarged accessible surface areas on SWCNTs called “DNA corona defects” (DCDs) which make “capture ssDNA” extended from DNA origami easy to adsorb on SWCNTs instead of traditional DNA hybridization strategy to achieve efficient positioning of SWCNTs on DNA origami boards.

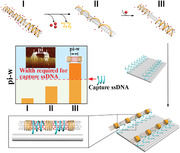

## 1 Introduction

The unique electronic, optical, and mechanical properties of single‐walled carbon nanotubes (SWCNTs) have made them ideal candidates for optoelectronic, catalytic, and sensing applications.^[^ ^1^ ^]^ The precise arrangement of SWCNTs with nanoscale resolution is required in many applications.^[^ ^1^, ^2^ ^]^ For example, the controllable spacing of SWCNTs facilitates high‐quality emission and absorption at the single‐photon level in photonics,^[^ ^2^ ^]^ and aligned SWCNTs significantly improve the gas detection sensitivity in electrode platforms.^[^ ^3^ ^]^ Traditional template modifications (represented by photolithography^[^ ^4^ ^]^) or dielectrophoresis^[^ ^5^ ^]^ approaches have been used to fabricate carbon nanotube (CNT) arrays; however, it remains challenging to arrange CNTs at the nanoscale with high efficiency.

DNA origami,^[^ ^6^ ^]^ as a milestone in DNA nanotechnology, formed by folding a long single‐stranded DNA with the help of many short staple strands, has been applied in precise positions of nanomaterials.^[^ ^7^ ^]^ Single‐stranded DNA (ssDNA) is a common dispersing agent for SWCNTs via wrapping on the surface due to the strong π–π stacking interactions between the aromatic nucleotides and the surface of SWCNTs.^[^ ^8^ ^]^ This wrapping DNA is called DNA corona,^[^ ^9^ ^]^ and the adsorption property enables the acquisition of DNA‐mediated SWCNT arrangement. DNA origami serves as a template to guide the alignment of SWCNTs by complementary hybridizations between DNA origami “capture strands” and “hanging ssDNA” on SWCNTs or adsorption of “capture strands” onto the surface of SWCNTs.^[^ ^10^ ^]^ However, the low assembly efficiency is still a problem that impedes the efficient arrangement of SWCNTs, limited by DNA hybridization efficiency or confined accessible surface of SWCNTs. Although some optimized strategies have been presented to improve assembly efficiency, these methods usually require multiple processing steps or DNA templates with specific structures.^[^ ^11^ ^]^ So, it is necessary to develop a simple and efficient strategy.

**Figure 1:**
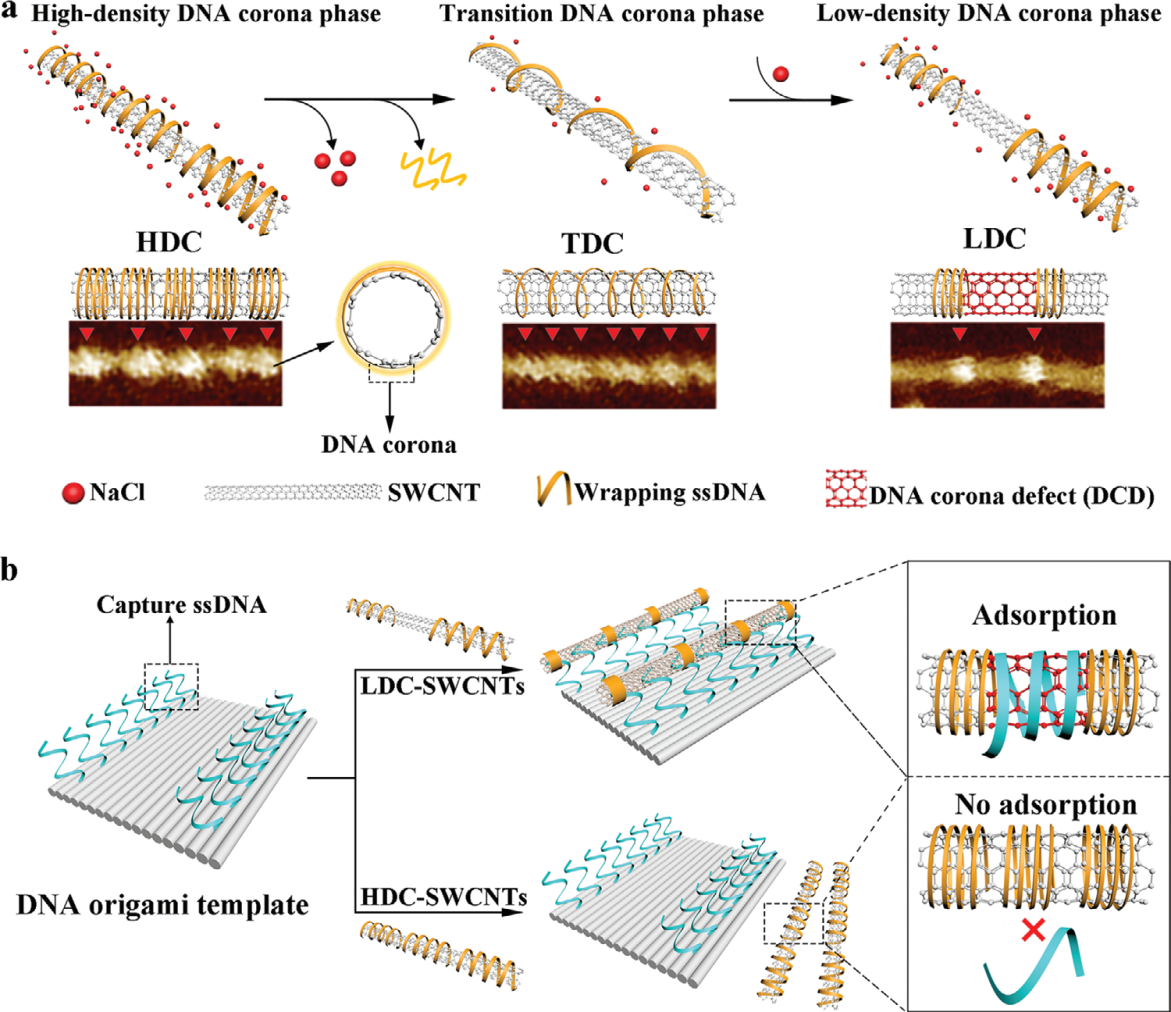
Schematic representation of the ionic strength‐mediated “DNA corona defects” (DCDs) strategy for efficient arrangement of SWCNTs on DNA origami template. a) DNA corona phase transition on SWCNT adjusted by solution ionic strength: high‐density DNA corona phase, HDC, with a compact and dense conformation; transition DNA corona phase, TDC, with a loose conformation; low‐density DNA corona phase, LDC, with a locally compact conformation releasing enlarged accessible surface on SWCNTs called DCDs. (b) Comparison diagram of LDC‐SWCNT and HDC‐SWCNT positioning on rectangular DNA origami template via “capture ssDNA” adsorption.

The wrapping process of DNA corona is usually performed at high salt concentrations to acquire high DNA coverages,^[^ ^12^ ^]^ which benefits the aqueous solubilization of SWCNTs but sacrifices the accessible surface area of SWCNTs. The DNA corona conformation on SWCNTs is sensitive to ionic strength determined by both the interactions in SWCNT‐DNA and the self‐stacking of DNA bases.^[^ ^13^ ^]^ In order to acquire the largest possible accessible surface while keeping good dispersibility, we developed an ionic strength‐mediated “DNA corona defects” (DCDs) strategy and applied it to improve the assembly efficiency of SWCNT on DNA origami (**Figure** **1**). Specifically, DNA strands adopt a compact conformation at high salt concentrations (Figure 1a, high‐density DNA corona phase, HDC) because of the enhanced self‐stacking of nucleobases. In contrast, ssDNA strands stretch along the SWCNTs to form a loose conformation at a low salt concentration (Figure 1a, transition DNA corona phase, TDC) dominated by interactions between SWCNT and ssDNA.^[^ ^9^ ^]^ By regulating the solution ionic strength, the DNA corona phase transitioned from an even‐distributed and loose conformation (TDC) to a locally compact conformation (Figure 1a, low‐density DNA corona phase, LDC). The released enlarged accessible surface of SWCNTs is called DCDs, which was distributed between adjacent DNA corona structures. The formed DCDs provided enlarged adsorption space for the “capture strands” extending from DNA origami, thus improving the assembling efficiency of SWCNTs on DNA origami. Efficient assembly supported the precise arranging of SWCNTs at the nanoscale on DNA origami templates with an average angular deviation of less than 5° (Figure 1b). Furthermore, a dual‐signal logic system based on the DNA origami template was fabricated using fluorescence quenching by SWCNTs. Our work provides a strategy to modulate the uneven DNA corona distribution on SWCNTs that generates localized charge distribution on the surface of SWCNTs, leading to exciton localization.^[^ ^14^ ^]^ It has potential applications for SWCNT‐based optoelectronic devices.

## 2 Results and Discussion

### 2.1 Ionic Strength‐Mediated DNA Corona Phase Transitions

**Figure 2:**
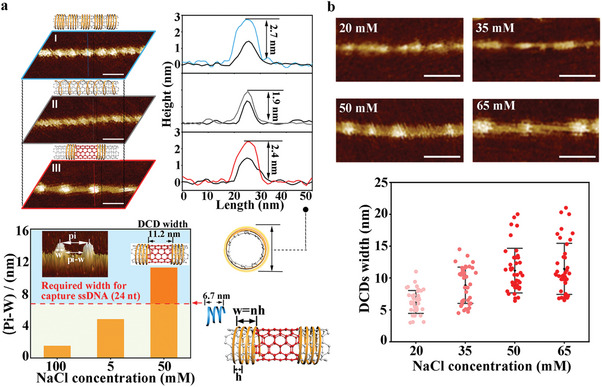
DNA corona phase transitions on the surface of SWCNTs are mediated by NaCl concentration. a) Local representative AFM images of SWCNTs exhibiting three DNA corona phases, with the statistical height of peaks and the value of pi‐w at three phases. Calculations indicate that the size of DCD (11.2 nm) is large enough for “capture ssDNA” adsorption (requiring 6.7 nm for 24‐nt). I, II, III correspond to HDC‐SWCNTs (100 mm NaCl), TDC‐SWCNTs (5 mm NaCl), and LDC‐SWCNTs (50 mm NaCl), respectively. Scale bars, 20 nm. b) Local representative AFM images and the statistics of DCD width on LDC‐SWCNTs at various NaCl concentrations (n = 41). Data are presented as mean ± SD. Scale bars, 20 nm.

According to the previous study, we obtained aqueous soluble SWCNTs with (GT)_15_ wrapping ssDNA at 100 mM NaCl solution (HDC, I).^[^ ^9^ ^]^ Next, the NaCl concentration was lowered to 5 mM (TDC, II) and then increased to 50 mM after removing the desorbed ssDNA (LDC, III). Atomic force microscopy (AFM) measurements and cross‐section analysis showed the structures of wrapping ssDNA on SWCNTs in the above three stages (**Figure** **2**a, upper). In the HDC and TDC phases, the wrapped DNA patterns were evenly and densely distributed along the entire SWCNTs. The height of DNA peaks decreased from HDC (≈2.7 nm) to TDC (≈1.9 nm), and the width narrowed from HDC (≈8.0 nm) to TDC (≈2.0 nm). At a high NaCl concentration (100 mm), wrapping DNA strands adopted compact conformations, making a pitch for the individual turns hard to distinguish by AFM tips. Hence, each height peak arose from each DNA molecule on the SWCNT, appearing as wider peaks. With the reduction of NaCl concentration (drop to 5 mm), the more elongation of ssDNA adsorbed on SWCNTs compelled partial wrapping ssDNA to desorb from SWCNTs. At this stage (TDC), the height peak arose from each turn of helically wrapped DNA. We conducted molecular weight interception filtration experiments to verify that a decrease in NaCl concentration can cause partial desorption of ssDNA from the surface of SWCNTs (Figure S1, Supporting Information).^[^ ^9^, ^15^ ^]^ After removing all the free ssDNA from the HDC‐SWCNT solution (100 mm), this stock solution was divided into two aliquots, followed by the dilution with 100 mm NaCl solution and water (final concentration of NaCl to 5 mm) respectively. The dilutions were incubated at room temperature overnight, and then centrifugal filtrations were performed to separate the free ssDNA for quantification. Results showed that the concentration of free ssDNA at the low salt concentration was significantly higher than that at the high salt concentration (from 162 to 20 nm). This indicates that a decrease in the salt concentration could cause partial ssDNA desorption from the surface of SWCNTs. Rising the NaCl concentration (50 mm) changed the elongational wrapping ssDNA back to a compact conformation with an increased peak width of up to ≈9.0 nm and improved height to ≈2.4 nm (LDC). Each peak arose from each DNA molecule while peaks were far from each other, releasing more exposed surface area on SWCNTs as DCDs. The monotonically decreased fluorescence intensity of ssDNA‐SWCNTs with NaCl concentration varying from 100 to 5 mm also supported the phase transition of DNA corona depending on solution ionic strength (Figure S2, Supporting Information). The width of each DCD is equal to the pitch (pi, the distance between the centers of adjacent DNA molecules) minus the width of the peak (w), which can be measured with AFM imaging. In our method, we obtained a pi‐w value of up to ≈11 nm (Figure 2a, below).

We adjusted the NaCl concentration to 20, 35, 50, and 65 mm at the LDC stage to investigate the effect of the NaCl concentration on the width of DCD. As shown in Figure 2b and Figure S3 (Supporting Information), the AFM characterization results indicated that the width of DCD gradually increased by raising the NaCl concentration until it reached a plateau of 50 mm. Given this, 50 mm NaCl was chosen as the final concentration at LDC in the following experiments. To test the influence of the DNA sequence length on the width of DCD, we selected (GT)_10_ and (GT)_20_ ssDNA as dispersing agents of SWCNTs to perform phase transition experiments of ionic strength‐mediated DNA corona, which was compared with the results of (GT)_15_ ssDNA. SWCNTs with (GT)_10_ ssDNA partially aggregated at low NaCl concentration (5 mm), which may be due to the relatively weak adsorption affinity between (GT)_10_ ssDNA and SWCNTs. For (GT)_20_ ssDNA, as shown in Figures S4 and S5 (Supporting Information), the statistical result of the DCD width was similar to that treated with (GT)_15_ ssDNA.

### 2.2 Assembly of SWCNTs on the Edges of DNA Origami with DCD Strategy

DCDs can serve as adsorption sites for “capture ssDNA” extended from DNA origami to enable the assembly of SWCNTs on DNA origami. “Capture ssDNA” is usually set between 20–30 nt for stable adsorption^[^ ^16^ ^]^ (here, 24‐nt ssDNA was chosen). Next, we need to know the occupied space on a SWCNT for a single 24‐nt ssDNA molecule at compact conformation, which can be calculated via the helical wrapping pitch (h) times the number of turns (n). The helical wrapping pitch (h) is approximately equal to that in HDC or LDC, which can be calculated from the Pythagorean Theorem; it is assumed that a cylinder with diameter d is formed via rolling a rectangle of diagonal l, where the length (l) of the diagonal makes exactly one helical turn around the cylinder: $L=\mathrm{nl}=n\sqrt{h^{2}+{\left(\pi d\right)}^{2}}$, where L is the total length of the ssDNA molecule and d is the diameter of SWCNTs (Figure S6).^[^ ^17^ ^]^ The helical wrapping pitch (h) for ssDNA at compact conformation is ≈2.0 nm, and the required width occupying an SWCNT for a single 24‐nt DNA molecule is ≈7.0 nm; details are shown in Supporting Information. Results revealed that each DCD (pi‐w in LDC) could support enough space for the adsorption of each 24‐nt DNA molecule (Figure 2a, below).

**Figure 3:**
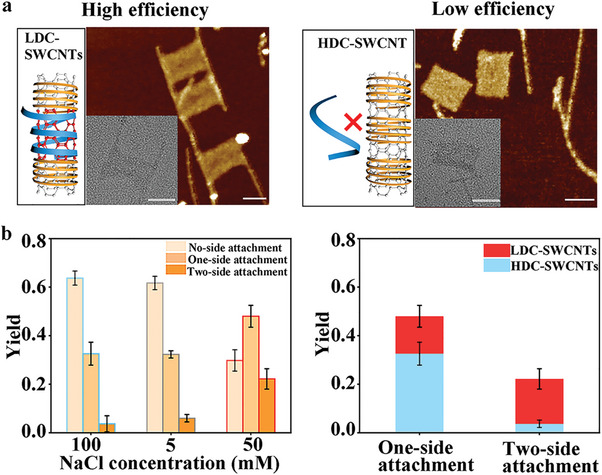
Assembly of SWCNTs on the edges of DNA origami with DCD strategy. a) A comparison of LDC‐SWCNT‐origami and HDC‐SWCNT‐origami using AFM and TEM images. Scale bars, 50 nm. b) Assembly yield analysis of HDC/TDC/LDC SWCNTs on DNA origami (n = 3). Data are presented as mean ± SD.

Here, the rectangular origami structure was used as an assembly template, extending 24‐nt ssDNA molecules from the two edges as “capture ssDNA”. SWCNTs were parallelly assembled on the two opposite sides via “capture ssDNA”. Representative AFM and transmission electron microscopy (TEM) images (**Figure** **3**a) demonstrate the comparison of assembly efficiency between LDC‐SWCNTs and HDC‐SWCNTs. Two‐side ligation (two sides of origami tile both attached with SWCNTs), one‐side ligation (only one side of origami tile attached with SWCNT), and 0‐side ligation (no attachment) complexes with distinct DNA wrapping configurations (HDC, TDC, and LDC) were counted and analyzed, as shown in Figure 3b, Figure S7, and Table S1 (Supporting Information), respectively. Results revealed that the yield for one‐side attachment increased from 33% (HDC‐SWCNTs) to 48% (LDC‐SWCNTs), while that for two‐side attachment elevated from 4% (HDC‐SWCNTs) to 22% (LDC‐SWCNTs). The edge staples of DNA origami were removed to release a partial scaffold strand as “capture ssDNA” to adsorb SWCNTs, which has been reported by others that the assembly efficiency of one‐side attachment is less than 30% and two‐side attachment is less than 10%.^[^ ^10c^ ^]^ We adopted a similar DNA template using a released partial scaffold strand as “capture ssDNA” to implement our strategy to further demonstrate the superiority of our method. Results revealed that the assembly efficiency of SWCNTs was observably higher in the LDC phase (50 mM NaCl) compared with that in HDC (100 mm NaCl) and TDC (5 mm NaCl) phases, as shown in Figures S8,S9, and Table S2 (Supporting Information). The yield for one‐side attachment increased from 33% (HDC‐SWCNTs) to 58% (LDC‐SWCNTs), while that for two‐side attachment elevated from 4% (HDC‐SWCNTs) to 10% (LDC‐SWCNTs).

### 2.3 Arrangement of SWCNTs on DNA Origami Surfaces with DCD Strategy

**Figure 4:**
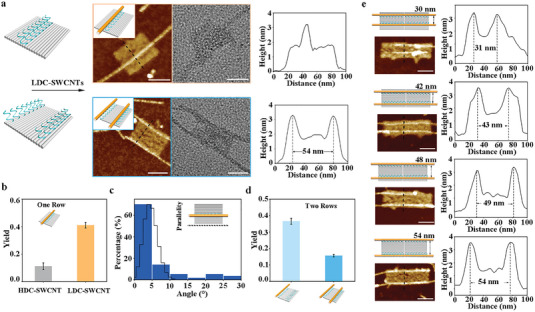
Arrangement of SWCNTs on DNA origami surfaces with DCD strategy. a) LDC‐SWCNTs positioning on the surface of DNA origami with one/two‐row “capture ssDNA” and the topography analysis from AFM and TEM images. b) Assembly yield analysis comparing HDC‐ and LDC‐SWCNTs on origami with a single row of “capture ssDNA” (n = 3). Data are presented as mean ± SD. c) Statistical analysis of the angle between the LDC‐SWCNT and the edge of the origami. d) LDC‐SWCNTs assembly yields on origami with two rows of “capture ssDNA” (n = 3). Data are presented as mean ± SD. e) Accurate arrangements of SWCNTs with spacing from 54 to 30 nm. Scale bars, 50 nm.

We tested the assembly efficiency of LDC‐SWCNTs on the surface of rectangular tiles to realize the precise arrangement of SWCNTs on the DNA origami template. One/Two rows of 24‐nt “capture ssDNA” strands parallel to the long side were extended from one face of the rectangle. Local representative AFM and TEM images showed the topological structures of the LDC‐SWCNT‐origami complexes (**Figure** **4**a). The histogram (yellow bar) displays the assembly yield (∼41%) of LDC‐SWCNT on the origami with one‐row “capture ssDNA” (Figure 4b; Figure S10, and Table S3, Supporting Information), which was significantly higher (≈4‐fold) than that of HDC‐SWCNT (gray bar). We analyzed the angular deviation of the assembled SWCNT with the designed orientation, which was aligned parallel to the long edge of the DNA origami. The statistical results showed that the average angular deviation was less than 5° (Figure 4c). Blue bars display the SWCNTs assembly yields on origami with two‐row “capture ssDNA” (≈16% for two‐attachment, ≈37% for one‐attachment, Figure 4d; Figure S11, and Table S4, Supporting Information). The measured separation between two SWCNTs was ≈54 nm, consistent with the design.

Next, the separation distance between two SWCNTs was precisely modulated by changing the distribution of “capture ssDNA” on origami. Origami dimers were built by connecting bridging strands to provide a larger template for SWCNTs arrangement (Figure S12 Supporting Information). Two rows of “capture ssDNA” strands were extended from the origami template with distances of 30, 42, 48, and 54 nm. Representative AFM images show the LDC‐SWCNT‐origami complexes consisting of the designs (Figure 4e), and statistics display that the average spacing deviation was ≈1 nm (Table S5).

### 2.4 Logic Gates based on DNA Origami Templates for SWCNTs

**Figure 5:**
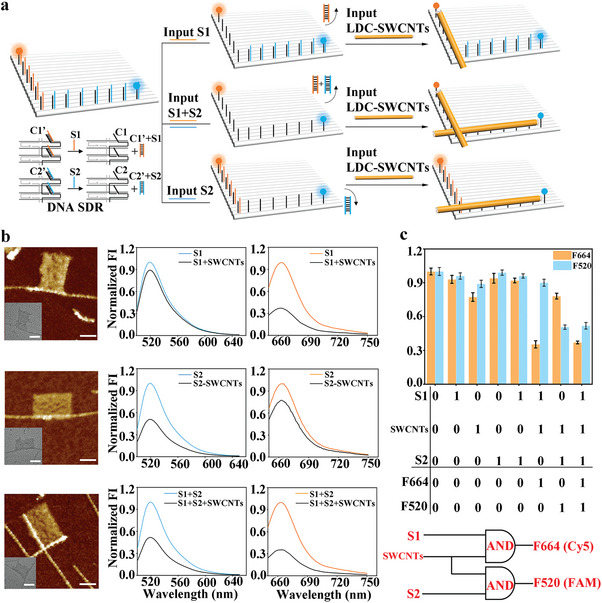
Logic gates based on DNA origami templates for SWCNTs. a) Schematic representation of a double‐signal logic system on DNA origami breadboard using SWCNTs‐induced fluorescence quenching. b) Representative AFM/TEM images of this logic system with three combinations of inputs (S1+LDC‐SWCNTs, S2+LDC‐SWCNTs, and S1+S2+LDC‐SWCNTs) and the corresponding observed fluorescence outputs. Scale bars, 40 nm. c) Circuit diagram and the statistical analysis of fluorescence intensity in response to different inputs (n = 3). Data are presented as mean ± SD.

The addressability of DNA origami makes it a breadboard for logic computing. As described in **Figure** **5**a, two vertical trajectories were designed with extended dsDNA strands from the surface of the rectangular origami tile. At the same time, FAM and Cy5 fluorophores were fixed at one end of each trajectory, respectively. Input fuel strands (S1) hybridized with C1’ and liberated C1 along the circuit line (parallel to the short edge of the origami breadboard) as “capture ssDNA”. Due to the strong interaction between ssDNA and SWCNTs compared with the weak affinity between dsDNA and SWCNTs,^[^ ^18^ ^]^ in the presence of fuel strands (S1), input LDC‐SWCNTs was positioned on origami. Excited Cy5 fluorophore was quenched by the located LDC‐SWCNTs. In the same way, input fuel strands S2 liberated capture ssDNA C2 in another line (parallel to the long edge of the origami breadboard) and captured LDC‐SWCNTs on this trajectory, which quenched excited FAM fluorophore. Input fuel strands S1 and S2, the two kinds of fluorophores‐Cy5 and FAM, were both quenched. Representative AFM and TEM images (Figure 5b, left; Figure S13, Supporting Information) show the locations of LDC‐SWCNTs on rectangular origami tiles as designed with various combinations of input signals (S1+LDC‐SWCNTs, S2+LDC‐SWCNTs, and S1+S2+LDC‐SWCNTs). The corresponding fluorescence curves (Figure 5b, right) demonstrate the correctness of the logic function of this double‐signal logic system. We measured the fluorescence value of the control group, including DNA origami solution without fuel strands and LDC‐SWCNT but diluting with an equal amount of buffer. The measured fluorescence value was set to 1 for the normalization of experimental data.^[^ ^19^ ^]^ The statistical analysis (Figure 5c) revealed the fluorescence emission response of this system to the input signals with eight possibilities via permutation and a combination of the three input signals (S1, S2, and LDC‐SWCNTs). The presence and absence of inputs were defined as “1” and “0,” while the outputs of normalized fluorescence intensity above and below the threshold (0.6) were defined as “0” and “1,” respectively. When only LDC‐SWCNTs were introduced, a small amount of LDC‐SWCNTs might be adsorbed to double‐stranded DNA (C1/ C1’ or C2/ C2’) on DNA origami. So, a slight decrease in fluorescence intensity was observed.^[^ ^20^ ^]^ Both S1/S2 and LDC‐SWCNTs as input signals displayed significant fluorescence decline at 664/520 nm, while input S1, S2, and LDC‐SWCNTs triggered fluorescence decline at both 664 and 520 nm. This, in line with the design, reveals the coexistence of S1 and LDC‐SWCNTs or S2 and LDC‐SWCNT built AND logic gates. SWCNT logic gates constructed on DNA origami templates can be used to create nanoscale optoelectronic circuits and devices with precise SWCNT arrangement and controllable responsiveness.

## 3 Conclusion

In summary, we demonstrated an ionic strength‐mediated “DNA corona defects” (DCDs) strategy to improve the assembly efficiency of SWCNTs on DNA origami. SWCNTs were located on DNA origami via “capture ssDNA” adsorption, displacing the traditional DNA hybridization strategy. High assembly efficiency was achieved via the formation of DCDs on SWCNTs, which provided enough space for “capture ssDNA” adsorption. Incorporating the programmability of DNA origami, SWCNTs were precisely arrayed in parallel lines with spacing at nanometer resolution. A double‐signal logic system on the origami board was built based on the SWCNT‐induced fluorescence quenching to further verify the efficient and accurate assembly of SWCNTs on DNA origami circuits by our approach. Furthermore, our method could achieve the controllable conformational adjustment of the DNA corona phase on SWCNTs. The uneven distribution of wrapping ssDNA carrying negative charges generates localized charge distribution on the surface of SWCNTs, providing traps for exciton and leading to exciton localization.^[^ ^14^ ^]^ Therefore, our developed DCD strategy provides a possible approach for adjusting the exciton energy variations and has potential for future applications in SWCNT‐DNA‐based electronics, photonics, or sensors.

## 4 Experimental Section

### Materials

All staple oligonucleotides were purchased from Sangon Biotech Co., Ltd. (Shanghai, China) and were used without further purification. M13mp18 single‐stranded DNA (7249) was purchased from New England Biolabs Inc. (Ipswich, MA, USA). SWCNTs were purchased from Nanjing XFNANO Materials Tech Co. Ltd. Other chemicals were purchased from Sinopharm and Sigma‐Aldrich.

### Wrapping SWCNTs with ssDNA

In a typical experiment, 0.5 mg SWCNT was dispersed in 600 µL DNA solution with 33 µm ssDNA and 100 mm NaCl. The sample was sonicated in an ice‐water bath environment for 30 min. After sonication, the SWCNTs were centrifugated at 16,000 g for 90 min at 4 °C to remove any undissolved substance. The supernatant, containing the dispersed SWCNTs, was collected and had a mass concentration range of 0.2‐0.4 mg mL^−1^. It was stored at 4 °C until further use.

### Purification of SWCNTs

Before each use, the stock solution was diluted and then purified to eliminate free ssDNA using the Amicon Ultra‐0.5 mL centrifugal filter (MWCO 100 kD). The NaCl concentration in the solution was maintained at 100 mM.

### Regulation of Ionic Strength of SWCNTs Solution

The NaCl concentration of the ssDNA‐SWCNT stock solution was reduced from 100 to 5 mm and incubated overnight at room temperature. After removing the ssDNA desorbed from the SWCNT surface using Amicon Ultra‐0.5 mL centrifugal filter (MWCO 100 kD), the NaCl concentration was restored to 50 mm and incubated overnight.

### Preparation of DNA Origami

To prepare DNA origami, M13mp18 scaffold, short DNA staple strands, and capture staples were mixed in 1×TAE/Mg^2+^ buffer (5 mm Tris, 1 mM EDTA, and 12.5 mm magnesium acetate, pH 8.0). The mixture was heated to 95 °C for 5 min in a thermal cycler and then allowed to cool down to 25°C at a rate of 0.1 °C every 10 s. Excess staples were then removed by washing with 500 µL 1×TAE/Mg^2+^ buffer (5 mM Tris, 1 mM EDTA, and 3 mm magnesium acetate, pH 8.0) four times using the Amicon Ultra‐0.5 mL centrifugal filter (MWCO 100 kD).

### DNA Origami‐SWCNTs Assembly

3 µL of purified DNA origami solution was mixed with 6 µL of SWCNTs solution (10 ug mL^−1^) under different DNA corona phases. The NaCl concentration of the mixed solution was adjusted to match that of the SWCNTs solution under the respective corona phases, and the mixture was incubated for 2 h at 33 °C.

### Logic Gates Based on DNA Origami Templates for SWCNTs

The DNA origami circuit, which included the “capture ssDNA” C1, C2, and their complementary sequences C2’, C1’, was fabricated through one‐pot annealing. Excess staples were then removed by washing with 500 µL 1×TAE/Mg^2+^ buffer (5 mm Tris, 1 mm EDTA, and 3 mm magnesium acetate, pH 8.0) four times using the Amicon Ultra‐0.5 mL centrifugal filter (MWCO 100 kD). In the logic calculations, fuel strands (S1 or S2) were added into the DNA circuit solution and incubated for 4 h to trigger a toehold‐mediated strand displacement reaction (SDR),^[^ ^18^ ^]^ releasing “capture ssDNA” C1 or C2 for the adsorption of the subsequently introduced LDC‐SWCNTs. The fluorescence values of the experimental groups were measured using an Edinburgh FLS980 spectrometer. In the control group, an equal amount of buffer was added to the DNA circuit solution without introducing fuel strands and LDC‐SWCNTs, and incubated for 4 h. The fluorescence value of the control group was set to one to normalize the fluorescence values of the experimental groups. Each experiment has been repeated three times.

### Transmission Electron Microscopy (TEM)

For TEM imaging, a 5 µL sample drop was adsorbed on a carbon film‐coated copper grid for 5 min. The remaining solution was wiped off with filter paper, and then a drop of double‐distilled water was added to the grid to remove excess salt. For negative staining, the sample was stained with 2% uranyl formate aqueous solution for 1 min, and the remaining solution was absorbed with filter paper.

### Atomic Force Microscopy (AFM)

For AFM imaging, 30 µL NiCl_2_ (10 mm) was deposited on freshly cleaved mica and left to adsorb for 10 min. The mica surface was washed with water and dried with nitrogen. Next, 3 µL of sample solution was deposited onto the mica and left for 3 min. Excess salt was removed using a drop of doubly distilled H_2_O, and the sample was dried with nitrogen. AFM imaging was performed under air tapping mode using a Multimode VIII AFM (Bruker, Inc.). A “SCANASYST‐AIR” model cantilever (Bruker) with a spring constant of 0.4 N m^−1^ and a tip radius of 2 nm was chosen for the measurements. The AFM images were analyzed using NanoScope analysis software.

### Fluorescence Spectroscopy Analysis

Prior to fluorescence measurement, SWCNT stock solutions were diluted to 50 ug mL^−1^ with different ionic strength buffers and incubated overnight at room temperature. Data were collected using an Edinburgh FLS980 spectrometer with an excitation wavelength of 650 nm. The excitation and emission wavelengths of fluorophores used in the logic circuit experiments were 480/520 nm (FAM) and 630/664 nm (Cy5). All fluorescence intensity data were normalized.

### Modeling and Calculation of DNA Corona Conformation Wrapped on the Surface of SWCNTs

AFM measurements and cross‐section analysis showed the structures of wrapping ssDNA on SWCNTs in three distinct stages. Each peak in the height profile of the SWCNT‐DNA complex potentially corresponds to a single turn of the DNA helix or a single DNA molecule wrapped around the SWCNT, depending on the conformation of the DNA corona. To determine the number of turns of a single ssDNA molecule wrapped around an SWCNT surface, it can be assumed that a cylinder with diameter *d* was formed via rolling a rectangle of diagonal *l*, the length (l) of the diagonal making exactly one helical turn around the cylinder (Figure S6, Supporting Information). The diagonal *l* can be calculated using the Pythagorean theorem:^[^ ^17a^ ^]^

(1) $$l=\sqrt{h^{2}{+(\pi d)}^{2}}$$

Where *h* is the helical wrapping pitch, and *d* is the average diameter of SWCNTs (1.4 nm). The total length (L) of ssDNA is equal to the length l of ssDNA wound in one turn multiplied by the number of turns n:

(2) $$L\;=\;\mathrm{nl}\;=\;n\sqrt{h^{2}{+(\pi d)}^{2}}$$

The total length of (GT)_15_ ssDNA, containing 30 nucleobases with an average nucleobase spacing of 0.676 nm, was ≈20.28 nm. At 100 mM NaCl, the self‐stacking force between nucleobases under high ionic strength causes ssDNA to adopt a compact conformation. Each peak value in the height profile represents a single ssDNA molecule. The product of n and h represents the peak width (w) of the winding segment along the surface of SWCNT.^[^ ^17b^ ^]^ The measured average peak width was 8 ± 1.4 nm, indicating that (GT)_15_ ssDNA was wrapped around the carbon nanotubes at a helical pitch of h  =  1.9 nm for *n*  =  4.2 turns using Equation (2). Similarly, at 50 mM NaCl, the measured average peak width was 9 ± 1.6 nm, and it was calculated that ssDNA was wrapped around carbon nanotubes at a helical pitch of h  =  2.2 nm for *n* =  4.1 turns. The capture ssDNA (GT)_12_ extended from the DNA origami contains 24 bases, and its total length *L* was ≈16.224 nm. The helical wrapping pitch (h) was approximately equal to that in HDC or LDC, with h being ≈2.0 nm. It was calculated that (GT)_12_ ssDNA wraps carbon nanotubes with *n*  =  3.4 turns, indicating that the required width occupying an SWCNT for a single 24‐nt DNA molecule was ≈6.7 nm.

### Statistical Analysis

All experimental data were recorded from at least three independently repeated samples and data were presented as mean ± standard deviation (SD).

## Conflict of Interest

The authors declare no conflict of interest.

## Supporting information

Supporting Information

## Data Availability

The data that support the findings of this study are available from the corresponding authors upon reasonable request.
