# Supplementary material for: Ionic Strength‐Mediated “DNA Corona Defects” for Efficient Arrangement of Single‐Walled Carbon Nanotubes
Source: Adv Sci (Weinh). 2024 Jan 17;11(15):2308532. doi: 10.1002/advs.202308532 (PMC11022692; doi:10.1002/advs.202308532)
Supplement: Supplementary file 1 — Supporting Information [file ADVS-11-2308532-s001.pdf]

ADVANCED SCIENCE
Open Access

Supporting Information

for *Adv. Sci.*, DOI 10.1002/advs.202308532

Ionic Strength-Mediated "DNA Corona Defects" for Efficient Arrangement of Single-Walled Carbon Nanotubes

Yuanyuan Luo, Na Wu, Liqiong Niu, Pengyan Hao, Xiaoya Sun, Feng Chen and Yongxi Zhao**

Supporting Information

Ionic Strength-Mediated "DNA Corona Defects" for Efficient Arrangement of Single-Walled Carbon Nanotubes

Yuanyuan Luo, Na Wu, Liqiong Niu, Pengyan Hao, Xiaoya Sun, Feng Chen and Yongxi Zhao**

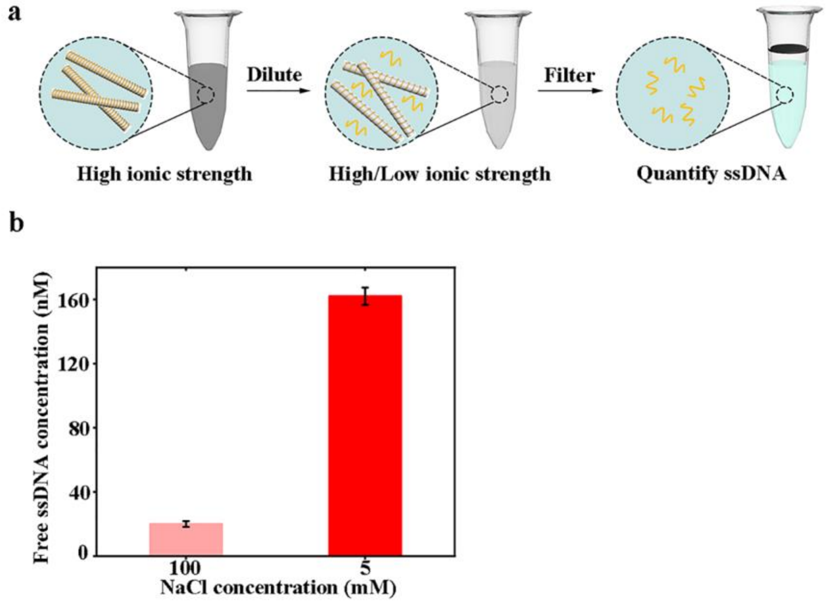

Figure S1. Quantifying free ssDNA desorbed from the surface of SWCNTs with NaCl concentration changes. a) Schematic diagram of the process for quantifying free ssDNA desorbed from the surface of SWCNTs. b) The histogram showed that the concentration of free ssDNA at low salt concentration is significantly higher than that at high salt concentration.

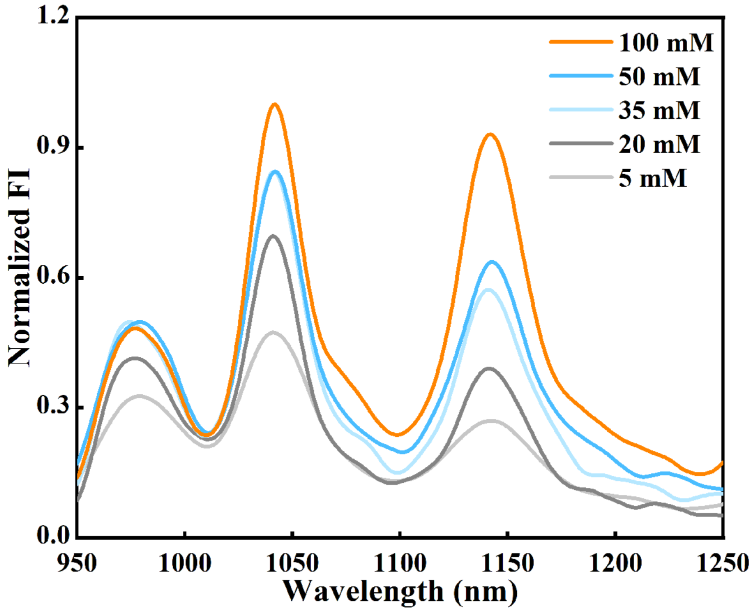

Figure S2. Normalized fluorescence intensity of ssDNA-SWCNTs at various NaCl concentrations. Monotonically decreased fluorescence intensity of ssDNA-SWCNTs with NaCl concentration varying from 100 mM to 5 mM, indicating that the surface coverage of DNA on SWCNTs varied with the ionic strength of the solution.

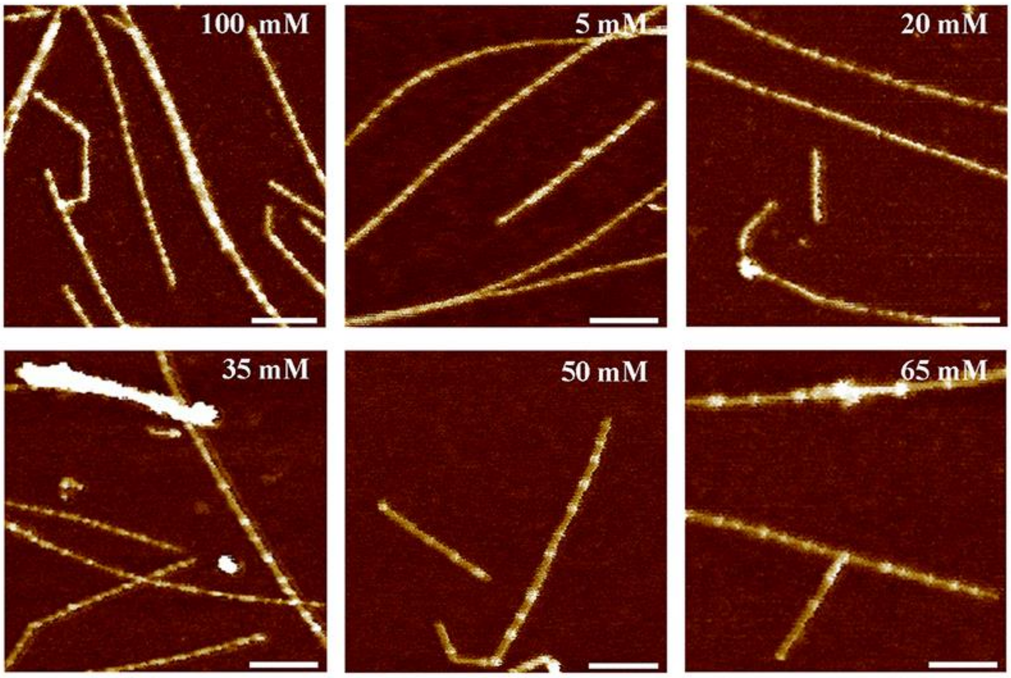

Figure S3. AFM images of $(GT)_{15}$ ssDNA-SWCNTs with different DNA corona phases. High density DNA corona phase, HDC (100 mM); transition DNA corona phase, TDC (5 mM); low density DNA corona phase, LDC (20 mM, 35 mM, 50 mM, and 65 mM). Scale bars: 50 nm.

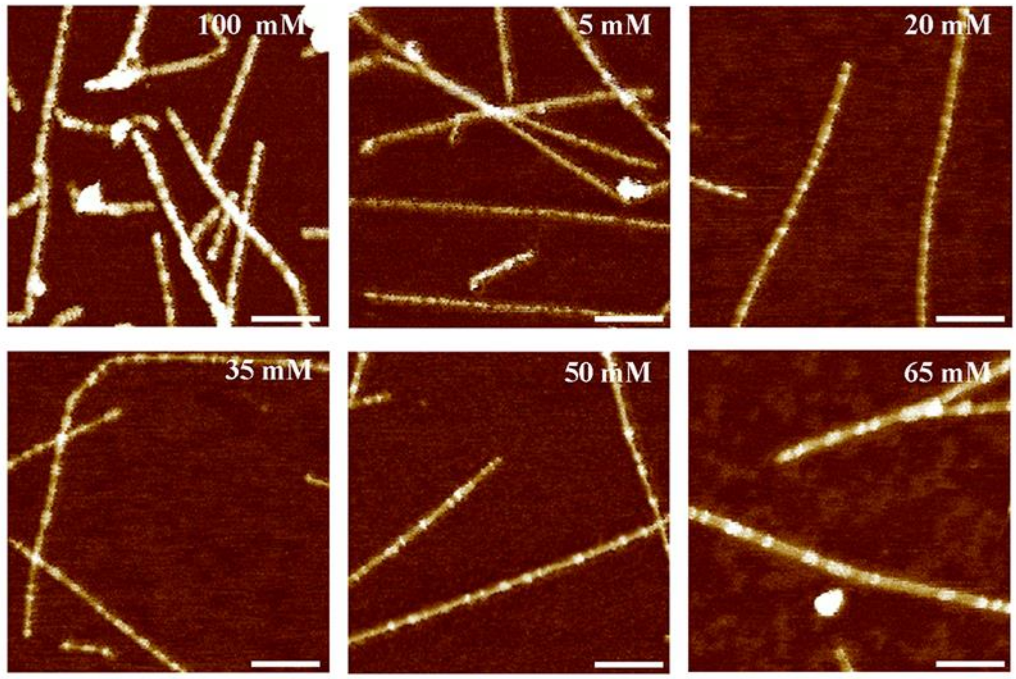

Figure S4. AFM images of $(GT)_{20}$ ssDNA-SWCNTs with different DNA corona phases. High density DNA corona phase, HDC (100 mM); transition DNA corona phase, TDC (5 mM); low density DNA corona phase, LDC (20 mM, 35 mM, 50 mM, and 65 mM). Scale bars: 50 nm.

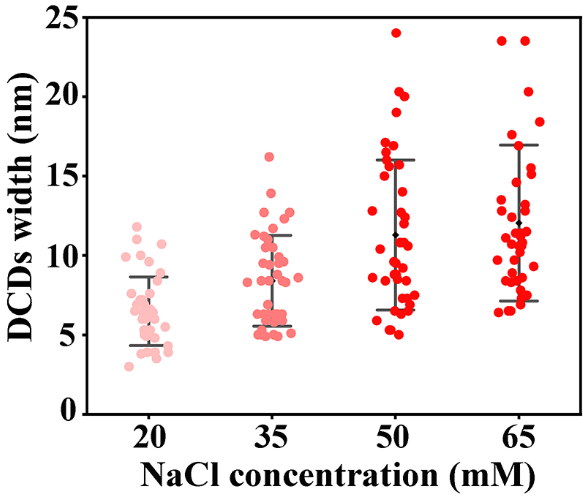

Figure S5. Statistical results of DCD width on SWCNTs with $(GT)_{20}$ ssDNA at different NaCl concentrations.

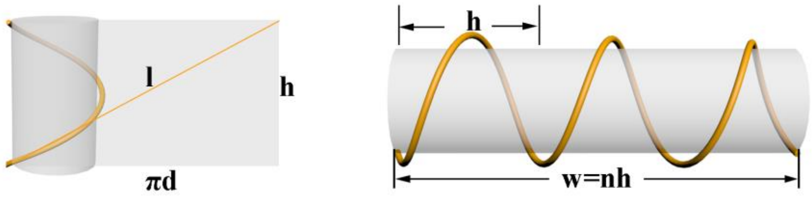

Figure S6. Schematic diagram of ssDNA winding model on SWCNT surface.

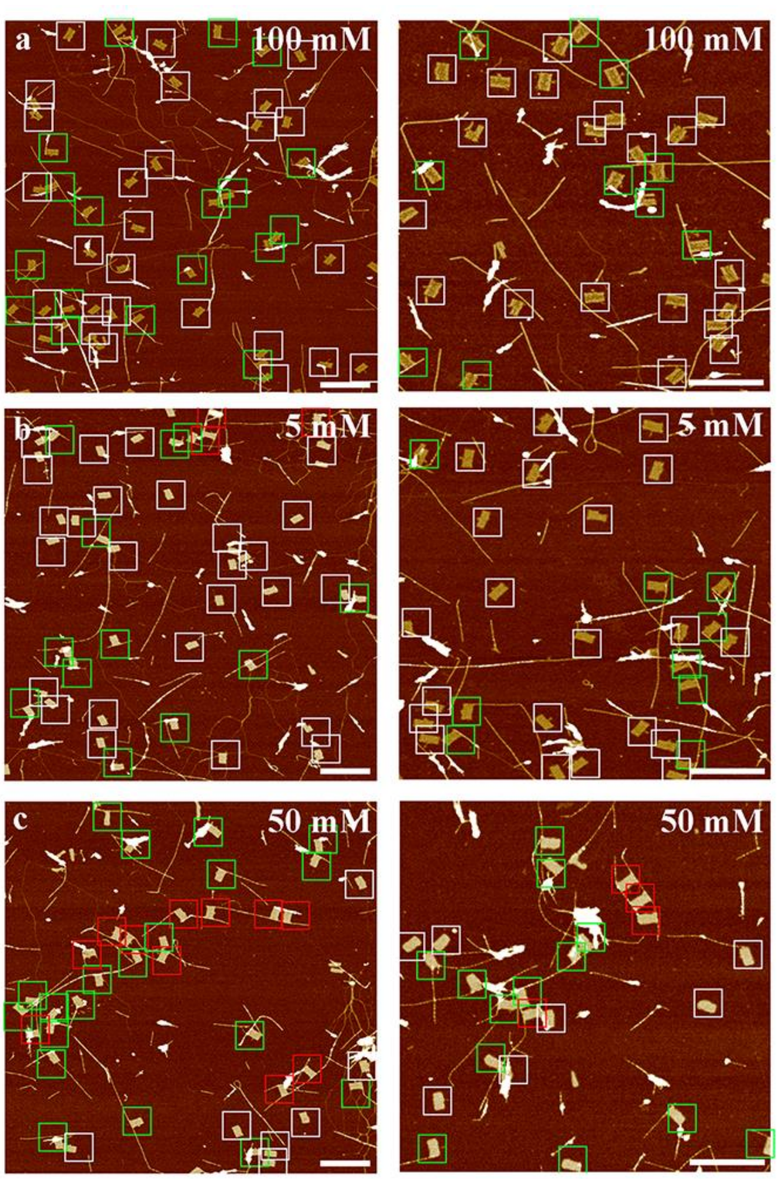

Figure S7. AFM images of the SWCNTs-origami complexes, in which DNA origami template with "capture ssDNA" extending from the edge staples; DNA wrapping SWCNTs including HDC-SWCNTs, TDC-SWCNTs, and LDC-SWCNTs. (a) 100 mM (HDC-SWCNTs; control group); (b) 5 mM (TDC-SWCNTs); (c) 50 mM (LDC-SWCNTs). No-side attachment: white solid boxes; one-side attachment: green solid boxes; two-side attachment: red solid boxes. Scale bars: 400 nm.

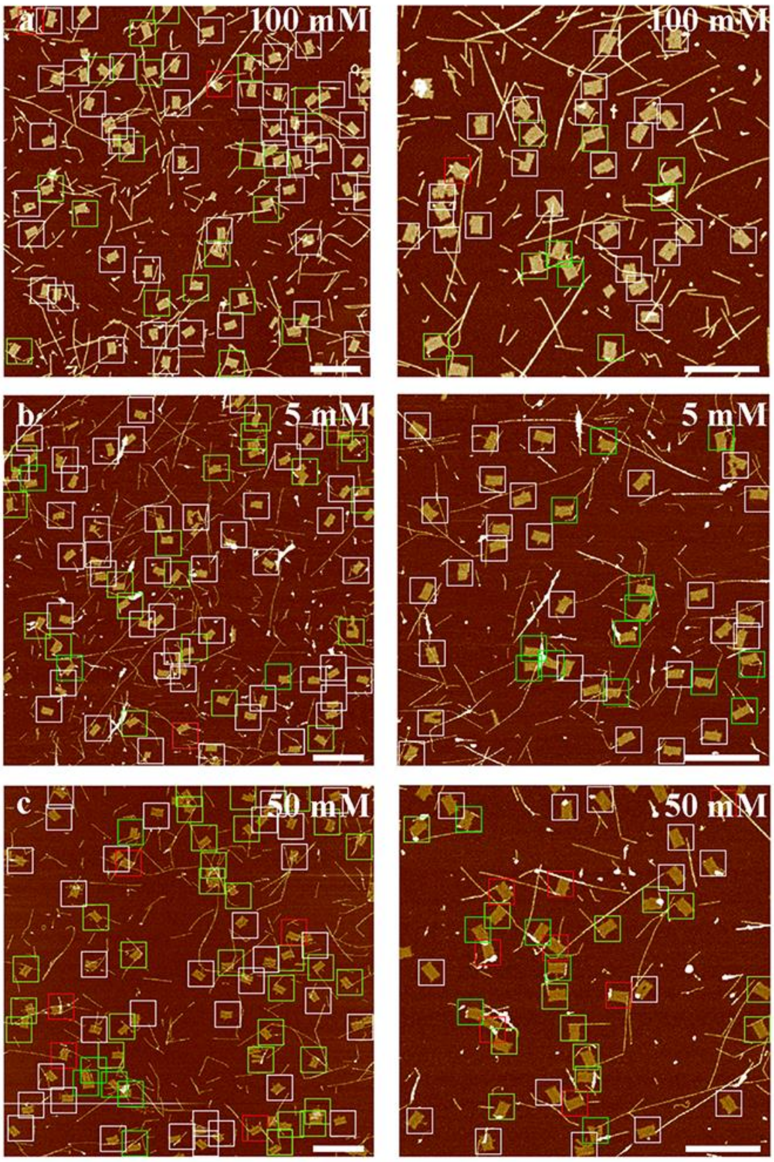

Figure S8. AFM images showcasing complexes formed between SWCNTs and DNA origami structures. The DNA origami templates employ the released scaffold loop strand as the "capture ssDNA" by removing the edge staples. DNA wrapping SWCNTs including HDC-SWCNTs, TDC-SWCNTs, and LDC-SWCNTs. (a) 100 mM (HDC-SWCNTs; control group); (b) 5 mM (TDC-SWCNTs); (c) 50 mM (LDC-

SWCNTs). No-side attachment: white solid boxes; one-side attachment: green solid boxes; two-side attachment: red solid boxes. Scale bars: 400 nm.

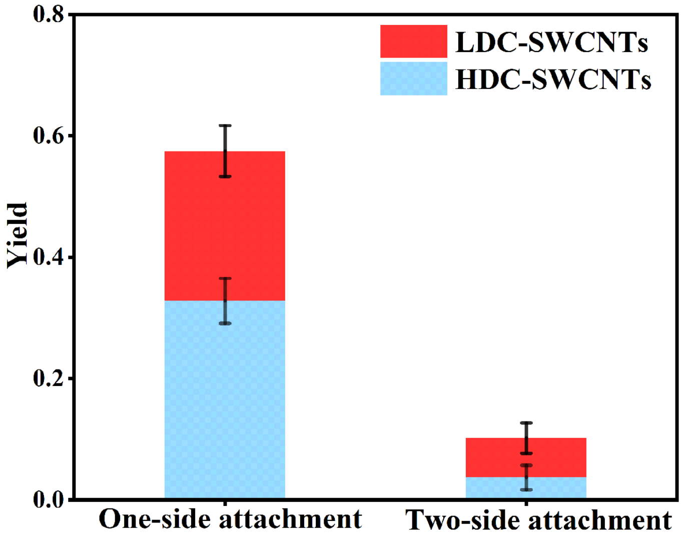

Figure S9. Assembly yield analysis of HDC /LDC SWCNTs on DNA origami (released scaffold loop strand as "capture ssDNA"). Compared with HDC-SWCNTs, the assembly efficiency of LDC-SWCNTs-origami is significantly improved.

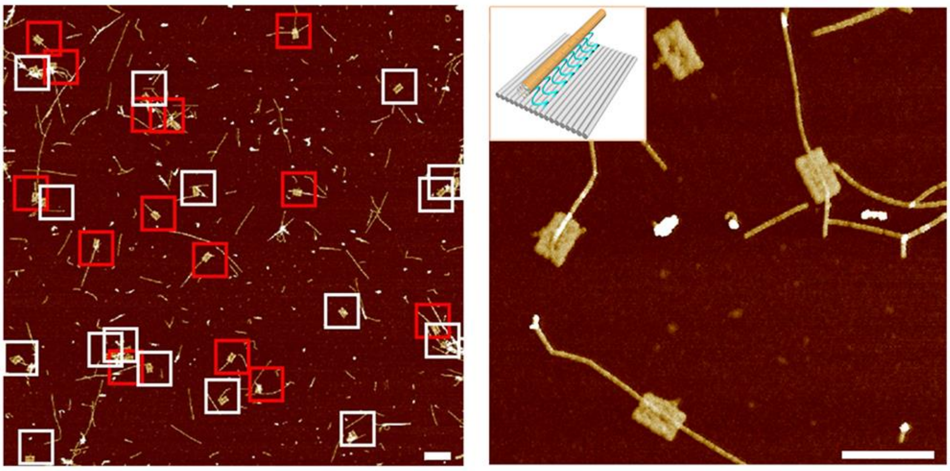

Figure S10. AFM images of single LDC-SWCNT positioning on the surface of DNA origami breadboard. Unassembled: white solid boxes; assembled: red solid boxes. Scale bars: 200 nm.

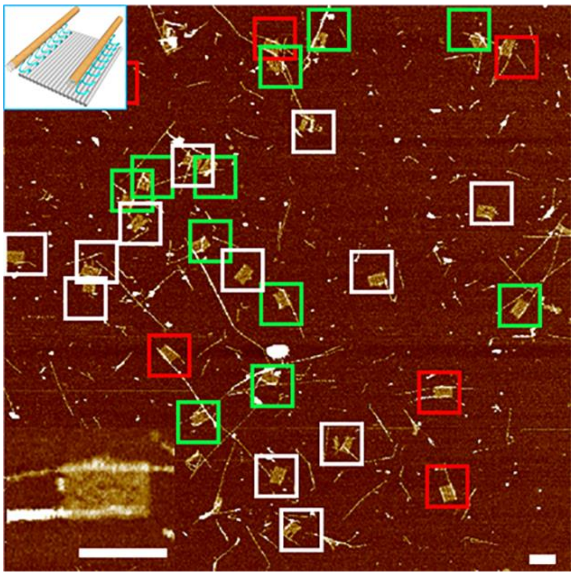

Figure S11. AFM images of two LDC-SWCNTs positioning on the surface of DNA origami breadboard. No SWCNT attachment: white solid boxes; one SWCNT attachment: green solid boxes; two SWCNTs attachment: red solid boxes. Scale bar: 100nm.

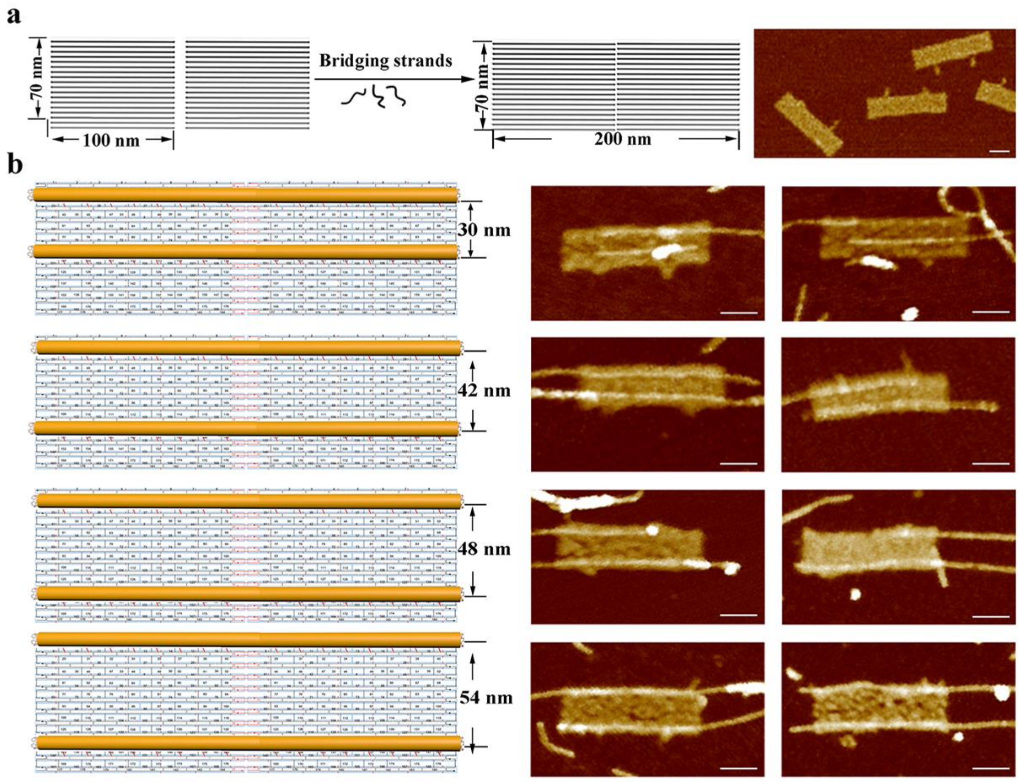

Figure S12. Origami dimers were built via the connection of bridging strands to provide a larger template for SWCNTs arrangement. a) Larger DNA origami breadboard built via bridging strands. b) Accurate arrangements of SWCNTs with spacing from 54 nm to 30 nm. Scale bars, 50 nm.

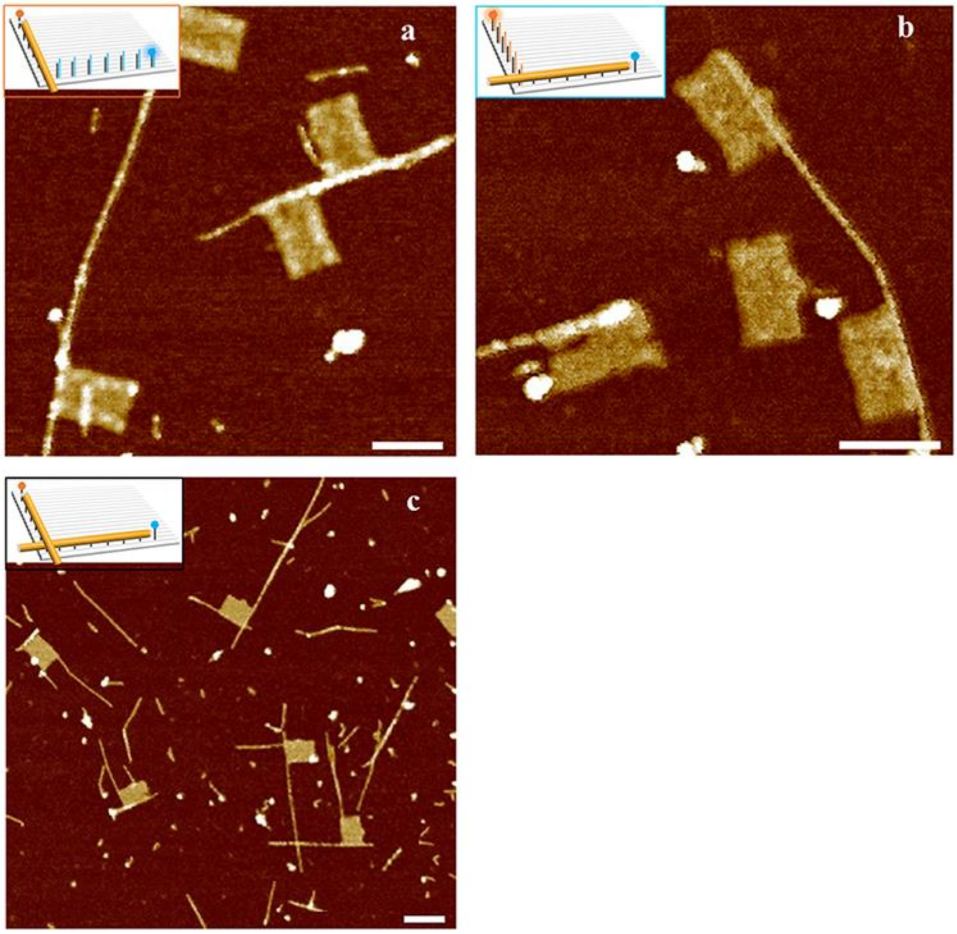

Figure S13. AFM images of logic system with three combinations of inputs. (a) S1+LDC-SWCNT. (b) S2+LDC-SWCNT. (c) S1+S2+LDC-SWCNT. Scale bar: 100 nm.

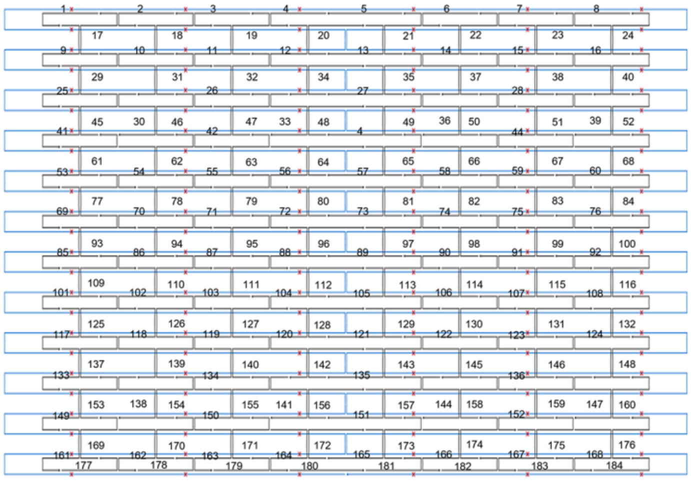

Figure S14. Schematic representation of DNA origami template using released scaffold loop strand as "capture ssDNA" by removing edge staples.

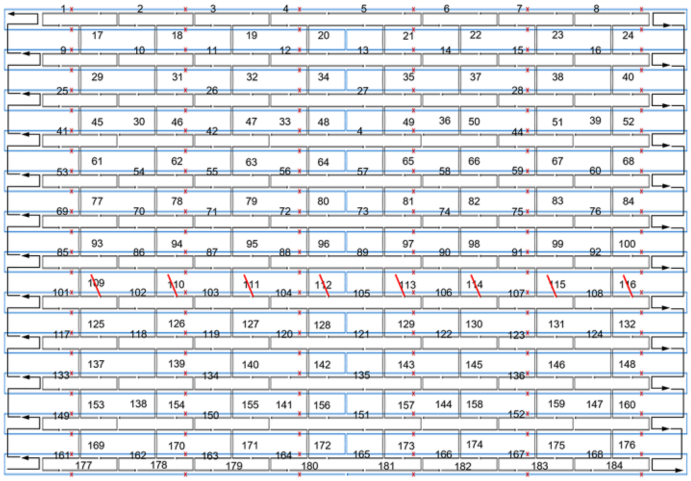

Figure S15. Schematic representation of one row of capture ssDNA protruding from the surface of DNA origami for the assembly of a single LDC-SWCNT, highlighted in red.

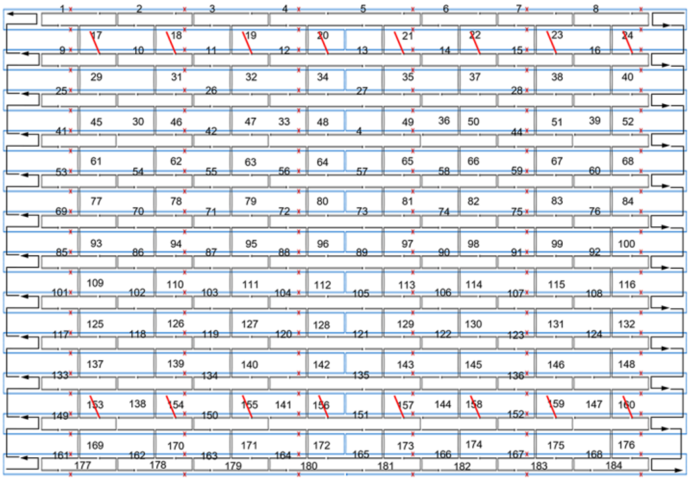

Figure S16. Schematic representation of two rows of capture ssDNA protruding from the surface of DNA origami for the assembly of LDC-SWCNTs, highlighted in red.

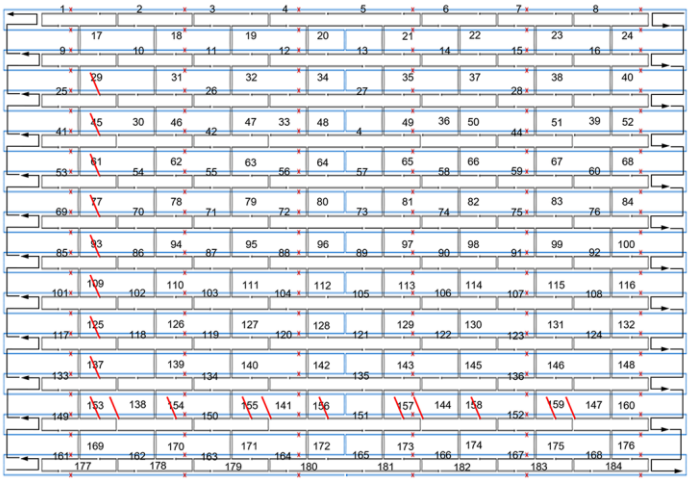

Figure S17. Schematic representation of DNA origami as a breadboard for logical computation, highlighted in red.

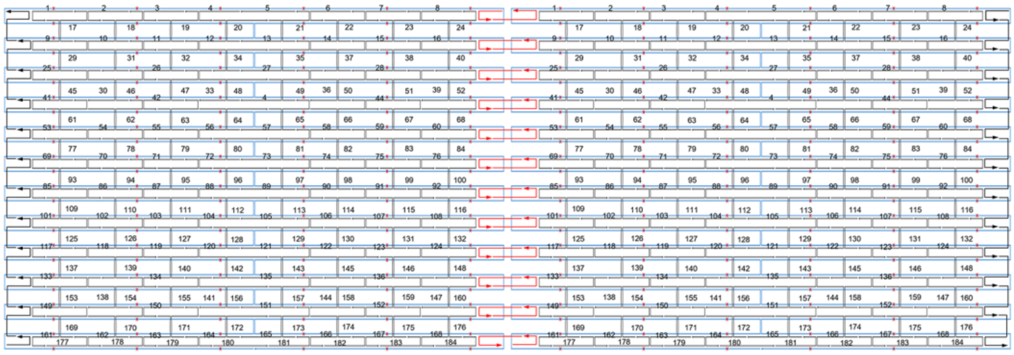

Figure S18. Schematic representation of DNA origami dimers, red highlighted bridging strands.

Table S1. Assembly yield analysis of SWCNTs using DNA origami template with two edges extending ssDNA as "capture ssDNA" under different salt concentrations.

Concentrations (mM)	No-side ligation (N)	Y (%)	One-side ligation (N)	Y (%)	Two-side ligation (N)	Y (%)
100	221	63.7 ± 2.9	113	32.6±4.7	13	3.7±3.3
5	206	61.7 ± 2.8	108	32.3±1.5	20	6.0±1.5
50	102	29.8 ± 4.4	164	48.0±4.5	76	22.2±4.2

Table S2. Assembly yield analysis of SWCNTs using DNA origami template with scaffold loop strand as "capture ssDNA" under different salt concentrations.

Concentrations (mM)	No-side ligation (N)	Y (%)	One-side ligation (N)	Y (%)	Two-side ligation (N)	Y (%)
100	256	63.5±3.0	132	32.8±3.7	15	3.7±2.0
5	266	66.5±3.2	124	31.0±2.5	10	2.5±1.2
50	132	32.3±2.9	235	57.5±4.2	42	10.2±2.5

Table S3. Yield of single HDC/LDC-SWCNT positioning on the surface of DNA origami breadboard.

	Ligation (N)	Y (%)
HDC-SWCNT	188	41.0±2.1
LDC-SWCNT	47	11.2±2.5

Table S4. Yield of two LDC-SWCNTs positioning on the surface of DNA origami breadboard.

Concentrations (mM)	No-side ligation (N)	Y (%)	One-side ligation (N)	Y (%)	Two-side ligation (N)	Y (%)
Two row SWCNTs	128	47.7±2.4	98	36.6±1.8	42	15.7±1.0

Table S5. Distance statistics of precise arrangement of SWCNTs with spacing from 54 nm to 30 nm.

Theoretical spacing (nm)	N	Measurements (nm)
30	25	30±0.91
42	23	41±1.02
48	25	49±1.01
54	22	54±1.03

DNA sequences

Capture sequences for anchoring SWCNTs on rectangular origami

DNA	Sequences (5'-3')
S1-029	GACCAACTAATGCCACTACGAAGGGGGTAGCAGTGTGTGTGTGTGTGTGTGTGT

S1-031	GCGCAGACAAGAGGCAAAAGAATCCCTCAGGTGTGTGTGTGTGTGTGTGT
S1-032	GACCTGCTCTTTGACCCCCAGCGAGGGAGTTAGTGTGTGTGTGTGTGTGTGT
S1-013	TTAGGATTGGCTGAGACTCCTCAATAACCGATGTGTGTGTGTGTGTGTGTGT
S1-035	CACCAGAAAGGTTGAGGCAGGTCATGAAAGGTGTGTGTGTGTGTGTGTGT
S1-037	CCACCCTCTATTCACAAACAAATACCTGCCTAGTGTGTGTGTGTGTGTGTGT
S1-038	GCCTCCCTCAGAATGGAAAGCGCAGTAACAGTGTGTGTGTGTGTGTGTGTGT
S1-040	AAATCACCTTCCAGTAAGCGTCAGTAATAAGTGTGTGTGTGTGTGTGTGT
S2-045	TACGTTAAAGTAATCTTGACAAGAACCGAACTGTGTGTGTGTGTGTGTGTGT
S2-046	TTATACCACCAAATCAACGTAACGAACGAGGTGTGTGTGTGTGTGTGTGT
S2-047	ATTACCTTTGAATAAGGCTTGCCCAAATCCGCGTGTGTGTGTGTGTGTGTGT
S2-027	TTGACAGGCCACCACCAGAGCCGCGATTTGTAGTGTGTGTGTGTGTGTGTGT
S2-049	CAGCAAAAGGAAACGTCACCAATGAGCCGCGTGTGTGTGTGTGTGTGTGT
S2-050	TCACCGACGCACCGTAATCAGTAGCAGAACCGGTGTGTGTGTGTGTGTGTGT

S2-051	GAAATTATTGCCTTTAGCGTCAGACCGGAACCGTGTGTGTGTGTGTGTGTGTGT
S2-052	ACCGATTGTCGGCATTTTCGGTCATAATCAGTGTGTGTGTGTGTGTGTGTGT
S3-125	ACAAACGGAAAAGCCCCAAAAACACTGGAGCAGTGTGTGTGTGTGTGTGTGTGT
S3-126	GCGAGTAAAAATATTTAAATTGTTACAAAGGTGTGTGTGTGTGTGTGTGTGT
S3-127	TGTAGCCATTAAAATTCGCATTAAATGCCGGAGTGTGTGTGTGTGTGTGTGTGT
S3-105	GTAATAAGTTAGGCAGAGGCATTTATGATATTGTGTGTGTGTGTGTGTGTGTGT
S3-129	TATAACTAACAAAGAACGCGAGAACGCCAAGTGTGTGTGTGTGTGTGTGTGT
S3-130	ACCTTTTTATTTTAGTTAATTTCATAGGGCTTGTGTGTGTGTGTGTGTGTGTGT
S3-131	GAATTTATTTAATGGTTTGAAATATTCTTACCGTGTGTGTGTGTGTGTGTGTGT
S3-132	CTTAGATTTAAGGCGTTAAATAAAGCCTGTGTGTGTGTGTGTGTGTGTGTGT
S4-153	TTAATGAACTAGAGGATCCCCGGGGGGTAACGGTGTGTGTGTGTGTGTGTGTGT
S4-154	TTCCAGTCGTAATCATGGTCATAAAAGGGGGTGTGTGTGTGTGTGTGTGTGT
S4-155	CACATTAAAATTGTTATCCGCTCATGCGGGCCGTGTGTGTGTGTGTGTGTGTGT

S4-135	AGAAAACAAAGAAGATGATGAAACAGGCTGCGGTGTGTGTGTGTGTGTGTGTGT
S4-157	ATTATCATTCAATATAATCCTGACAATTACGTGTGTGTGTGTGTGTGTGTGT
S4-158	GCGGAACATCTGAATAATGGAAGGTACAAAATGTGTGTGTGTGTGTGTGTGTGT
S4-159	ATTTTAAAATCAAAATTATTTGCACGGATTCGGTGTGTGTGTGTGTGTGTGTGT
S4-160	CTCGTATTAGAAATTGCGTAGATACAGTACGTGTGTGTGTGTGTGTGTGTGT
S5-169	CTCCAACGCAGTGAGACGGGCAACCAGCTGCAGTGTGTGTGTGTGTGTGTGTGT
S5-170	TGGAACAACCGCCTGGCCCTGAGGCCCGCTGTGTGTGTGTGTGTGTGTGTGT
S5-171	GCCCGAGAGTCCACGCTGGTTTGCAGCTAACTGTGTGTGTGTGTGTGTGTGTGT
S5-151	GCAATTCACATATTCCTGATTATCAAAGTGTAGTGTGTGTGTGTGTGTGTGTGT
S5-173	ACCTTGCTTGGTCAGTTGGCAAAGAGCGGAGTGTGTGTGTGTGTGTGTGTGT
S5-174	AGCCAGCAATTGAGGAAGGTTATCATCATTTTGTGTGTGTGTGTGTGTGTGTGT
S5-175	TTAACACCAGCACTAACAACTAATCGTTATTAGTGTGTGTGTGTGTGTGTGTGT
S5-176	CAGAAGATTAGATAATACATTTGTCGACAAGTGTGTGTGTGTGTGTGTGTGT

Sequences of double-signal logic system based on DNA origami

DNA	Sequences (5'-3')
C1-169	CTCCAACGCAGTGAGACGGGCAACCAGCTGCAGTGTGTGTGTGTGTGTGT
C1-153	TTAATGAACTAGAGGATCCCCGGGGGGTAACGTATTATTATTATTATTATTAT
C1-137	CCAGGGTTGCCAGTTTGAGGGGACCCGTGGGATATTATTATTATTATTATTAT
C1-125	ACAAACGGAAAAGCCCCAAAAACACTGGAGCATATTATTATTATTATTATTAT
C1-109	AACAAGAGGGATAAAAATTTTTAGCATAAAGCTATTATTATTATTATTATTAT
C1-093	TAAATCGGGATTCCCAATTCTGCGATATAATGTATTATTATTATTATTATTAT
C1-077	CTGTAGCTTGACTATTATAGTCAGTTCATTGATATTATTATTATTATTATTAT
C1-061	ATCCCCCTATACCACATTCAACTAGAAAAATCTATTATTATTATTATTATTAT
C1-045	TACGTTAAAGTAATCTTGACAAGAACCGAACTTATTATTATTATTATTATTAT
C2-138	ATTAAGTTTACCGAGCTCGAATTCGGGAAACCTGTCGTGCGTGTGTGTGTGTGTGTGT
C2-170	TGGAACAACCGCCTGGCCCTGAGGCCCGCTGTGTGTGTGTGTGTGTGT

C2-171	GCCCGAGAGTCCACGCTGGTTTGCAGCTAACTGTGTGTGTGTGTGTGTGTGT
C2-141	GCGATCGGCAATTCCACACAACAGGTGCCTAATGAGTGGTGTGTGTGTGTGTGTG T
C2-151	GCAATTCACATATTCCTGATTATCAAAGTGTAGTGTGTGTGTGTGTGTGTGT
C2-173	ACCTTGCTTGGTCAGTTGGCAAAGAGCGGAGTGTGTGTGTGTGTGTGTGT
C2-144	ATTCATTTTTGTTTGGATTATACTAAGAAACCACCAGAAGTGTGTGTGTGTGTGTGTG T
C2-174	AGCCAGCAATTGAGGAAGGTTATCATCATTTTGTGTGTGTGTGTGTGTGTGT
C2-175	TTAACACCAGCACTAACAACTAATCGTTATTAGTGTGTGTGTGTGTGTGTGT
FAM-147	AACAATAACGTAAAACAGAAATAAAAATCCTTTGCCCGAATTGTGTGTGTGTGTGTGT GTGT-FAM
Cy5-029	GACCAACTAATGCCACTACGAAGGGGGTAGCATTTATTATTATTATTATTATTAT-Cy5
C1’	CGACTATAATAATAATAATAATAATA
S1	TATTATTATTATTATTATTATAGTCG
C2’	TCCTAACACACACACACACACACAC

S2	GTGTGTGTGTGTGTGTGTGTTAGGA

Sequences of rectangular origami staple strands

Number	Sequences (5'-3')
001	AGAAAGGAACAACTAAAGGAATTCAAAAAAA
002	ACAACTTTCAACAGTTTCAGCGGATGTATCGG
003	TAAATGAATTTTCTGTATGGGATTAATTTCTT
004	TCTAAAGTTTTGTCGTCTTTCCAGCCGACAA
005	TCCACAGACAGCCCTCATAGTTAGCGTAACGA
006	TCACCAGTACAAACTACAACGCCTAGTACCAG
007	AGGAACCCATGTACCGTAACACTTGATATAA
008	CCACCCTCATTTTCAGGGATAGCAACCGTACT
009	AGGCTCCAGAGGCTTTGAGGACACGGGTAA
010	TTTATCAGGACAGCATCGGAACGACACCAACCTAAAACGAGGTCAATC
011	AAACAGCTTTTTGCGGGATCGTCAACACTAAA

012	TGACAACTCGCTGAGGCTTGCATTATACCAAGCGCGATGATAAA
013	TTAGGATTGGCTGAGACTCCTCAATAACCGAT
014	GCGGATAACCTATTATTCTGAAACAGACGATTGGCCTTGAAGAGCCAC
015	GTATAGCAAACAGTTAATGCCCAATCCTCA
016	CAGGAGGTGGGGTCAGTGCCTTGAGTCTCTGAATTTACCGGGAACCAG
017	ACGGCTACAAAAGGAGCCTTTAATGTGAGAAT
018	CAGCGAAACTTGCTTTCGAGGTGTTGCTAA
019	AAGGCCGCTGATACCGATAGTTGCGACGTTAG
020	ATATTCGGAACCATCGCCCACGCAGAGAAGGA
021	TATTAAGAAGCGGGGTTTTGCTCGTAGCAT
022	TTTCGGAAGTGCCGTCGAGAGGGTGAGTTTCG
023	GCCCGTATCCGGAATAGGTGTATCAGCCCAAT
024	GTTTTAACTTAGTACCGCCACCCAGAGCCA
025	AATACGTTTGAAAGAGGACAGACTGACCTT
026	ACACTCATCCATGTTACTTAGCCGAAAGCTGC

027	TTGACAGGCCACCACCAGAGCCGCGATTTGTA
028	TTAAAGCCAGAGCCGCCACCCTCGACAGAA
029	GACCAACTAATGCCACTACGAAGGGGGTAGCA
030	ATAAGGGAACCGGATATTCATTACGTCAGGACGTTGGGAA
031	GCGCAGACAAGAGGCAAAAGAATCCCTCAG
032	GACCTGCTCTTTGACCCCCAGCGAGGGAGTTA
033	TTGTGTCGTGACGAGAAACACCAAATTTCAACTTTAAT
034	TCATCGCCAACAAAGTACAACGGACGCCAGCA
035	CACCAGAAAGGTTGAGGCAGGTCATGAAAG
036	CACCCTCAGAAACCATCGATAGCATTGAGCCATTTGGGAA
037	CCACCCTCTATTCACAAACAAATACCTGCCTA
038	GCCTCCCTCAGAATGGAAAGCGCAGTAACAGT
039	AGCCACCACTGTAGCGCGTTTTCAAGGGAGGGAAGGTAAA
040	AAATCACCTTCCAGTAAGCGTCAGTAATAA
041	CATCAAGTAAAACGAACTAACGAGTTGAGA

042	TCATTCAGATGCGATTTTAAGAACAGGCATAG
043	GCAAGGCCTCACCAGTAGCACCATGGGCTTGA
044	TCAAGTTTCATTAAAGGTGAATATAAAAGA
045	TACGTTAAAGTAATCTTGACAAGAACCGAACT
046	TTATACCACCAAATCAACGTAACGAACGAG
047	ATTACCTTTGAATAAGGCTTGCCCAAATCCGC
048	GATGGTTTGAACGAGTAGTAAATTTACCATTA
049	CAGCAAAAGGAAACGTCACCAATGAGCCGC
050	TCACCGACGCACCGTAATCAGTAGCAGAACCG
051	GAAATTATTGCCTTTAGCGTCAGACCGGAACC
052	ACCGATTGTCGGCATTTTCGGTCATAATCA
053	TTTAGGACAAATGCTTTAAACAATCAGGTC
054	ATGCAGATACATAACGGGAATCGTCATAAATAAAGCAAAG
055	TAAGAGCAAATGTTTAGACTGGATAGGAAGCC
056	CGTTTACCAGACGACAAAGAAGTTTTGCCATAATTCGA

057	TTATTACGAAGAACTGGCATGATTGCGAGAGG
058	CGTAGAAAATACATACCGAGGAAACGCAATAAGAAGCGCA
059	AACGCAAAGATAGCCGAACAAACCCTGAAC
060	GTTTATTTTGTCACAATCTTACCGAAGCCCTTTAATATCA
061	ATCCCCCTATACCACATTCAACTAGAAAAATC
062	AATACTGCCCAAAAGGAATTACGTGGCTCA
063	AATAGTAAACACTATCATAACCCTCATTGTGA
064	CTTTTGCAGATAAAAACCAAAATAAAGACTCC
065	ATACCCAACAGTATGTTAGCAAATTAGAGC
066	AAGGAAACATAAAGGTGGCAACATTATCACCG
067	AAGTAAGCAGACACCACGGAATAATATTGACG
068	AATAGCTATCAATAGAAAATTCAACATTCA
069	TTTACCCCAACATGTTTTAAATTTCCATAT
070	CGGATTGCAGAGCTTAATTGCTGAAACGAGTA
071	CGAAAGACTTTGATAAGAGGTCATATTTCGCA

072	GCTTCAATCAGGATTAGAGAGTTATTTTCA
073	AGAGAGAAAAAAATGAAAATAGCAAGCAAACT
074	TTAGACGGCCAAATAAGAAACGATAGAAGGCT
075	AAAGTCACAAAATAAACAGCCAGCGTTTTA
076	GAGAGATAGAGCGTCTTTCCAGAGGTTTTGAA
077	CTGTAGCTTGACTATTATAGTCAGTTCATTGA
078	GATGGCTTATCAAAAAGATTAAGAGCGTCC
079	TTGCTCCTTTCAAATATCGCGTTTGAGGGGGT
080	CCAACAGGAGCGAACCAGACCGGAGCCTTTAC
081	TTAACGTCTAACATAAAAACAGGTAACGGA
082	ATCCCAATGAGAATTAACTGAACAGTTACCAG
083	GCCAGTTAGAGGGTAATTGAGCGCTTTAAGAA
084	ACGCTAACACCCACAAGAATTGAAAATAGC
085	AACAGTTTTGTACCAAAAACATTTTATTTC
086	GATTTAGTCAATAAAGCCTCAGAGAACCCTCA

087	AATGGTCAACAGGCAAGGCAAAGAGTAATGTG
088	TTTGGGGATAGTAGTAGCATTAAAAGGCCG
089	CCAATAGCTCATCGTAGGAATCATGGCATCAA
090	TATCCGGTCTCATCGAGAACAAGCGACAAAAG
091	GCGAACCTCCAAGAACGGGTATGACAATAA
092	GCCTTAAACCAATCAATAATCGGCACGCGCCT
093	TAAATCGGGATTCCCAATTCTGCGATATAATG
094	AAATTAAGTTGACCATTAGATACTTTTGCG
095	TAAATCATATAACCTGTTTAGCTAACCTTTAA
096	TTCTACTACGCGAGCTGAAAAGGTTACCGCGC
097	TTTTATTTAAGCAAATCAGATATTTTTTGT
098	GTACCGCAATTCTAAGAACGCGAGTATTATTT
099	CTTATCATTCCCGACTTGCGGGAGCCTAATTT
100	TGTAGAAATCAAGATTAGTTGCTCTTACCA
101	AACGCAAAATCGATGAACGGTACCGGTTGA

102	TATATTTTGTCATTGCCTGAGAGTGGAAGATT
103	TAGGTAAACTATTTTTGAGAGATCAAACGTTA
104	GAGACAGCTAGCTGATAAATTAATTTTTGT
105	GTAATAAGTTAGGCAGAGGCATTTATGATATT
106	GTAAAGTAATCGCCATATTTAACAAAACTTTT
107	ACAACATGCCAACGCTCAACAGTCTTCTGA
108	GTTTATCAATATGCGTTATACAAACCGACCGT
109	AACAAGAGGGATAAAAATTTTTAGCATAAAGC
110	GCTATCAGAAATGCAATGCCTGAATTAGCA
111	GAGGGTAGGATTCAAAAGGGTGAGACATCCAA
112	CAACCGTTTCAAATCACCATCAATTCGAGCCA
113	CATGTAATAGAATATAAAGTACCAAGCCGT
114	AATTGAGAATTCTGTCCAGACGACTAAACCAA
115	AGTATAAAGTTCAGCTAATGCAGATGTCTTTC
116	TTAGTATCACAATAGATAAGTCCACGAGCA

117	TAATCAGCGGATTGACCGTAATCGTAACCG
118	GTATAAGCCAACCCGTCGGATTCTGACGACAGTATCGGCCGCAAGGCG
119	ATATTTTGGCTTTCATCAACATTATCCAGCCA
120	TAAATCAAAATAATTCGCGTCTCGGAAACCAGGCAAAGGGAAGG
121	ATCGCAAGTATGTAAATGCTGATGATAGGAAC
122	TCAAATATAACCTCCGGCTTAGGTAACAATTTCATTTGAAGGCGAATT
123	CCTAAATCAAAATCATAGGTCTAAACAGTA
124	GTGATAAAAAGACGCTGAGAAGA1GATAACCTTGCTTCTGTTCGGGAGA
125	ACAAACGGAAAAGCCCCAAAAACACTGGAGCA
126	GCGAGTAAAAATATTTAAATTGTTACAAAG
127	TGTAGCCATTAAAATTCGCATTAAATGCCGGA
128	GCCATCAAGCTCATTTTTTAACCACAAATCCA
129	TATAACTAACAAAGAACGCGAGAACGCCAA
130	ACCTTTTTATTTTAGTTAATTTCATAGGGCTT
131	GAATTTATTTAATGGTTTGAAATATTCTTACC

132	CTTAGATTTAAGGCGTTAAATAAAGCCTGT
133	TGCATCTTTCCCAGTCACGACGGCCTGCAG
134	GCTTTCCGATTACGCCAGCTGGCGGCTGTTTC
135	AGAAAACAAAGAAGATGATGAAACAGGCTGCG
136	CATAAATCTTTGAATACCAAGTGTTAGAAC
137	CCAGGGTTGCCAGTTTGAGGGGACCCGTGGGA
138	ATTAAGTTTACCGAGCTCGAATTCGGGAAACCTGTCGTGC
139	GATGTGCTTCAGGAAGATCGCACAATGTGA
140	TCTTCGCTGCACCGCTTCTGGTGCGGCCTTCC
141	GCGATCGGCAATTCCACACAACAGGTGCCTAATGAGTG
142	CAACTGTTGCGCCATTCGCCATTCAAACATCA
143	CTGAGCAAAAATTAATTACATTTTGGGTTA
144	ATTCATTTTTGTTTGGATTATACTAAGAAACCACCAGAAG
145	CGCGCAGATTACCTTTTTTAATGGGAGAGACT
146	CCTGATTGCAATATATGTGAGTGATCAATAGT

147	AACAATAACGTAAAACAGAAATAAAAATCCTTTGCCCGAA
148	CTTTTACAAAATCGTCGCTATTAGCGATAG
149	GTCGACTTCGGCCAACGCGCGGGGTTTTTC
150	CTGTGTGATTGCGTTGCGCTCACTAGAGTTGC
151	GCAATTCACATATTCCTGATTATCAAAGTGTA
152	CTACCATAGTTTGAGTAACATTTAAAATAT
153	TTAATGAACTAGAGGATCCCCGGGGGGTAACG
154	TTCCAGTCGTAATCATGGTCATAAAAGGGG
155	CACATTAAAATTGTTATCCGCTCATGCGGGCC
156	AAGCCTGGTACGAGCCGGAAGCATAGATGATG
157	ATTATCATTCAATATAATCCTGACAATTAC
158	GCGGAACATCTGAATAATGGAAGGTACAAAAT
159	ATTTTAAAATCAAAATTATTTGCACGGATTCG
160	CTCGTATTAGAAATTGCGTAGATACAGTAC
161	TTTTCACTCAAAGGGCGAAAAACCATCACC

162	AGCTGATTGCCCTTCAGAGTCCACTATTAAAGGGTGCCGT
163	AGCAAGCGTAGGGTTGAGTGTTGTAGGGAGCC
164	CCCAGCAGGCGAAAAATCCCTTATAAATCAAGCCGGCG
165	TCAATATCGAACCTCAAATATCAATTCCGAAA
166	TCAACAGTTGAAAGGAGCAAATGAAAAATCTAGAGATAGA
167	CTTTAGGGCCTGCAACAGTGCCAATACGTG
168	AGATTAGAGCCGTCAAAAAACAGAGGTGAGGCCTATTAGT
169	CTCCAACGCAGTGAGACGGGCAACCAGCTGCA
170	TGGAACAACCGCCTGGCCCTGAGGCCCGCT
171	GCCCGAGAGTCCACGCTGGTTTGCAGCTAACT
172	TCGGCAAATCCTGTTTGATGGTGGACCCTCAA
173	ACCTTGCTTGGTCAGTTGGCAAAGAGCGGA
174	AGCCAGCAATTGAGGAAGGTTATCATCATTTT
175	TTAACACCAGCACTAACAACTAATCGTTATTA
176	CAGAAGATTAGATAATACATTTGTCGACAA

177	CAAATCAAGTTTTTTGGGGTCGAAACGTGGA
178	AAAGCACTAAATCGGAACCCTAATCCAGTT
179	CCCGATTTAGAGCTTGACGGGGAAAAAGAATA
180	AACGTGGCGAGAAAGGAAGGGAAACCAGTAA
181	TAAAAGGGACATTCTGGCCAACAAAGCATC
182	ACCCTTCTGACCTGAAAGCGTAAGACGCTGAG
183	GCACAGACAATATTTTTGAATGGGGTCAGTA
184	CTTTAATGCGCGAACTGATAGCCCCACCAG
